# Halides as versatile anions in asymmetric anion-binding organocatalysis

**DOI:** 10.3762/bjoc.17.145

**Published:** 2021-09-01

**Authors:** Lukas Schifferer, Martin Stinglhamer, Kirandeep Kaur, Olga García Macheño

**Affiliations:** 1 Organic Chemistry Institute, Westfälische-Wilhelms University Münster, Correnstraße 36, 48149 Münster, Germany

**Keywords:** anion binding, asymmetric catalysis, halide anions, hydrogen donors, noncovalent interactions

## Abstract

This review intends to provide an overview on the role of halide anions in the development of the research area of asymmetric anion-binding organocatalysis. Key early elucidation studies with chloride as counter-anion confirmed this type of alternative activation, which was then exploited in several processes and contributed to the advance and consolidation of anion-binding catalysis as a field. Thus, the use of the halide in the catalyst–anion complex as both a mere counter-anion spectator or an active nucleophile has been depicted, along with the new trends toward additional noncovalent contacts within the HB-donor catalyst and supramolecular interactions to both the anion and the cationic reactive species.

## Introduction

**Figure 1:**
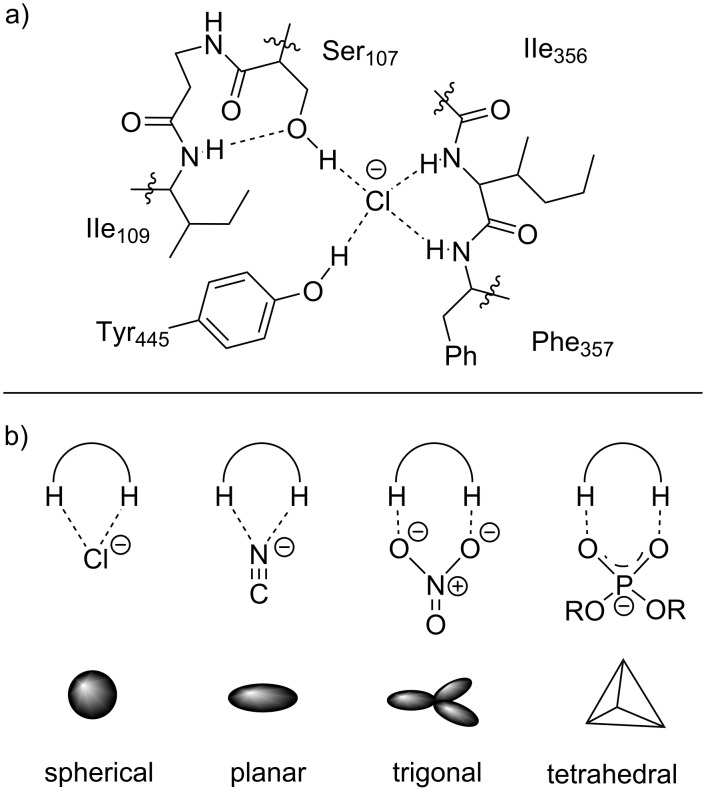
a) Binding interactions in the chloride channel of *E. coli.* and b) examples of chloride, cyanide, nitrate and phosphate anions with their respective topology.

Halogens and the respective anionic halides occupy an essential role in natural and chemical processes [1–4]. While in chemical syntheses halogens are often regarded as surrogates for further functionalization, their role in natural and physiological processes is much more diverse. One of these processes is the ability of large complex molecules and enzymes to recognize halide anions via hydrogen bonds in aqueous media [5]. Amongst others, the regulation of membrane potentials is one of such applications, in which the transport of chloride anions is facilitated by noncovalent hydrogen bonding interactions (Figure 1a) [6]. Noncovalent interactions are in fact one of the essential factors for the molecular recognition in enzymatic reactions, especially anionic species [7]. Even though initial reports of nonenzymatic halide recognition date back to the 1960s [8], strategies to exploit this ability for synthetic or catalytic purposes were vastly disregarded in the following decades [9]. This relies on the fact that it is highly challenging to design small molecule catalysts that resemble anion-binding properties of enzymes. Hence, a major challenge of small organic receptors to mimic nature’s capability of binding to the targeted anions resides in the supramolecular properties of enzymes and co-factors to form exact matching binding cavities. In this context, halides offer an advantage over various other anionic species because their spherical topology reduces the number of possible isomers or complexes upon interaction with the receptor. As a consequence, predictable cavity sizes based on the employed halide allows for easier targeting of the small receptor molecule and, thus, reducing the need for complexity compared to enzymes or co-factors. Conversely, a multitude of geometries may need to be considered for anions with linear, coplanar, trigonal or tetrahedral topologies (Figure 1b) [5,10].

However, following the advances in anion coordination and supramolecular chemistry [7–11], this field of research has attracted more attention within the past two decades. Immense efforts were made to identify small molecules that are able to productively bind anions via noncovalent hydrogen bonding, from which cationic receptors have often proven more efficient [9,12]. A breakthrough in the field of anion binding towards its application in catalysis was achieved with the findings that neutral (thio)urea derivatives are potent anion receptors due to their ability to bind anions of various topologies, including the spherical halides [10]. The key to hydrogen bonding of the halide anion resides in the polarized N–H bonds of these (thio)urea units, which have since served as a benchmark in the design and development of anion receptor catalysts [12–14]. Consequently, other synthetic anion receptors have been developed in the past decades, all based on polarized hydrogen bond motifs. While commonly based on N–H bonds [15–18], also polarized O–H [19–20] and even C–H [21–22] bond-based systems have been realized. As a consequence of the importance and increasing attention of this field, there are already a few reviews on anion-binding catalysis implying different types of anions [10,15,23–29]. However, in this review, we aim at providing an overview of the evolution of anion-binding catalysis by focusing on the key role of halides as decisive anions for the development of the concepts and implementation of natural principles of anion recognition by small molecule catalysts.

## Review

### Hydrogen bonding to neutral substrates or anion binding?

**Figure 2:**
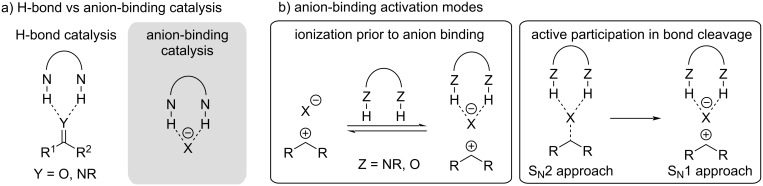
a) H-bond vs anion-binding catalysis and b) activation modes in anion-binding catalysis.

In the early stages of anion-binding-catalysis development, some reactions might have potentially been mistaken to be hydrogen-bond catalyzed [15,23]. While both catalyses are closely related by making use of hydrogen-bond interactions as the directing noncovalent force, they can be distinguished by the type of substrate that is bound to and activated by the catalyst (Figure 2a). In H-bond catalysis, neutral substrates such as carbonyl compounds are coordinated to the H-bond catalyst, whereas anion-binding catalysis relies on the formation of an ion pair by binding to the counter-anion of an ionic substrate. The ionization of the corresponding substrate can either occur before the coordination to the anion or the catalyst itself directly participates in the ionization step by an anion abstraction-type process (Figure 2b). In the latter approach, the C–X bond cleavage can then either follow a S_N_1 or S_N_2 pathway.

For enantioselective purposes, solvation of the ion pair is crucial for obtaining high stereoinduction. While more polar solvents give solvent-separated or solvent-shared ion pairs – in which the components have their own solvent shells –, nonpolar solvents are more likely to lead to contact-ion pairs. As such, the cation and anion are in closer proximity as one solvent shell is shared. If a chiral catalyst binds then to the anion, a chiral contact-ion pair can be formed, which is necessary for the transfer of the chiral information to the product. As a consequence, most of the reported methods embracing enantioselective anion-binding catalysis rely on the use of nonpolar solvents such as ethers or aromatic compounds.

### Pioneering work

**Scheme 1:**
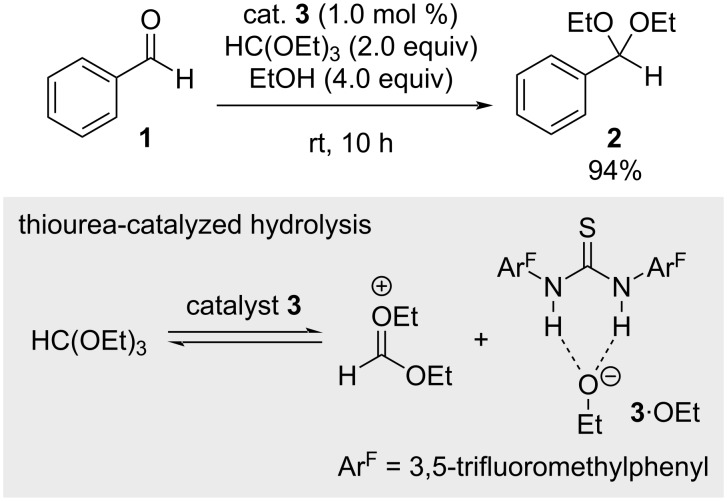
First proposed anion-binding mechanism in the thiourea-catalyzed acetalization of benzaldehyde.

The concept of anion-binding catalysis was first penned by Schreiner et al. in 2006, who realized the acetalization of benzaldehyde (**1**) with a thiourea catalyst (**3**, Scheme 1) [30–31]. They proposed the reaction to proceed via thiourea-catalyzed orthoester hydrolysis, leading to the formation of a catalyst-bound alkoxide species (**3**·OEt) that is then able to attack the benzaldehyde for product **2** formation.

**Scheme 2:**
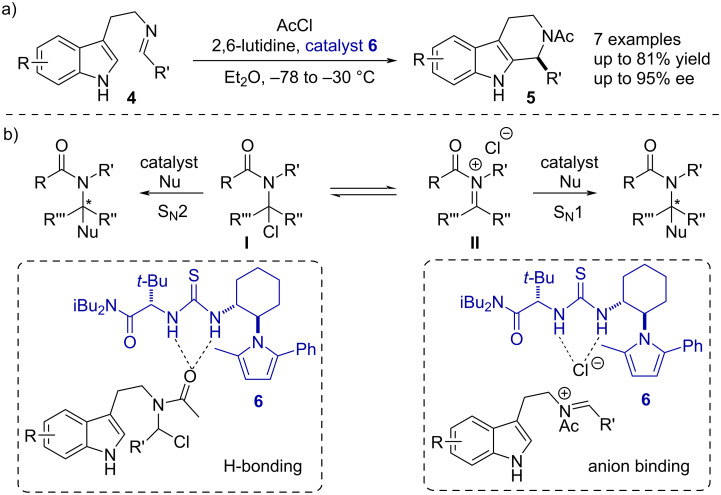
a) Thiourea-catalyzed enantioselective acyl-Pictet–Spengler reaction of tryptamine-derived imines **4**. b) Equilibrium between the ionic (S_N_1-type mechanism) and neutral form (S_N_2-type reaction). The key intermediates for the respective binding modes are displayed in the boxes.

However, it took some time until the scientific community started considering and taken cognizance of the potential of this type of activation mode in catalysis. In this regard, Jacobsen and co-workers reported in 2004 an asymmetric Pictet–Spengler reaction of tryptamine-derived imines **4** in the presence of acetyl chloride and 2,6-lutidine, where the chiral thiourea catalyst **6** was employed to enable good yields and enantioselectivities (Scheme 2a) [32]. The initial motivation of their first studies revolved around hydrogen bond donor catalysts and their application in *N*-acyliminium ion reactions. At this point, the mechanistic proposal, albeit speculative, was based on the hypothesis that neutral chloroamide structures **I** were the reactive intermediates in the reaction. Under this premise, H-bonding to the carbonyl group was proposed as the binding mode of the catalyst and the reaction to proceed via a S_N_2-type mechanism (Scheme 2b, left). Not considered at that time was the anion-binding pathway through the iminium chloride salt **II**, which would proceed via a S_N_1-type mechanism (Scheme 2b, right).

**Scheme 3:**
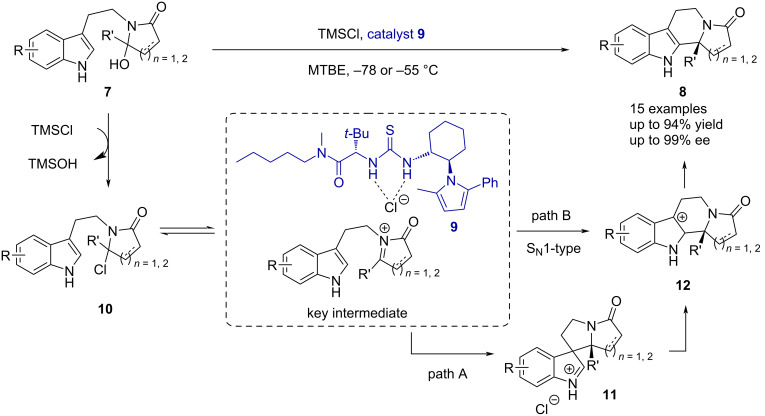
Proposed mechanism of the thiourea-catalyzed enantioselective Pictet–Spengler reaction of hydroxylactams **7**. First provided evidence of anion binding instead of carbonyl hydrogen bonding.

However, based on the freshly coined concept of anion-binding activation [30–31] and as the exact interaction mode of the catalyst remained elusive, Jacobsen’s group focused their attention towards mechanistic studies of thiourea-catalyzed reactions. In 2007, they reported a Pictet–Spengler cyclization reaction of succinimide and glutarimide-derived hydroxylactams **7** (Scheme 3) [33]. This system was designed in a way that key experimental observations could be made to analyze whether a S_N_1 or S_N_2-type mechanism takes place. A strong dependence of the enantioselectivity on the counterion and solvent was observed and, therefore, a S_N_1-type mechanism was concluded. Furthermore, their studies proved that an ion pair is required for the reaction to proceed and, most importantly, that the thiourea catalyst **9** interacts with the chloride of the *N*-acyliminium ion as opposed to the carbonyl group.

**Scheme 4:**
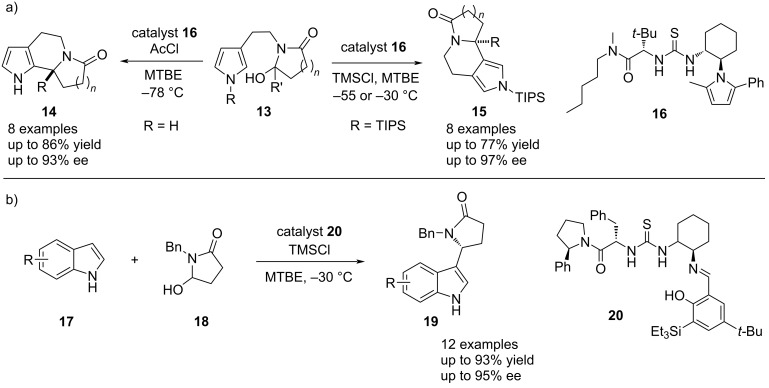
a) Thiourea-catalyzed intramolecular Pictet–Spengler-type cyclization of hydroxylactam-derived *N*-acyliminium chlorides and b) thiourea-catalyzed intermolecular hydroxy lactam-derived *N*-acyliminium chlorides with indoles.

Based on this concept, the applicability of *N*-acyliminium chlorides in thiourea-catalyzed anion-binding reactions was further explored. In 2008, an intramolecular asymmetric Pictet–Spengler-type cyclization reaction with pyrrole derivatives **13** was reported. The authors were not only able to control the enantioselectivity, but this system also allowed the control over regioselectivity (C2 vs C4 cyclization) through alteration of the *N*-substituent of the pyrrole substrate and the acylating reagent (Scheme 4a) [34]. This example showcases that next to the counterion, the acylating group can have a major influence on these types of reactions. The first thiourea-catalyzed asymmetric intermolecular reaction with *N*-acyliminium chlorides was then also realized by the same group in 2009. Therein, nucleophilic addition of indoles **17** to the *N*-acyliminium chlorides was achieved with excellent enantiomeric excess (Scheme 4b) [35].

**Scheme 5:**
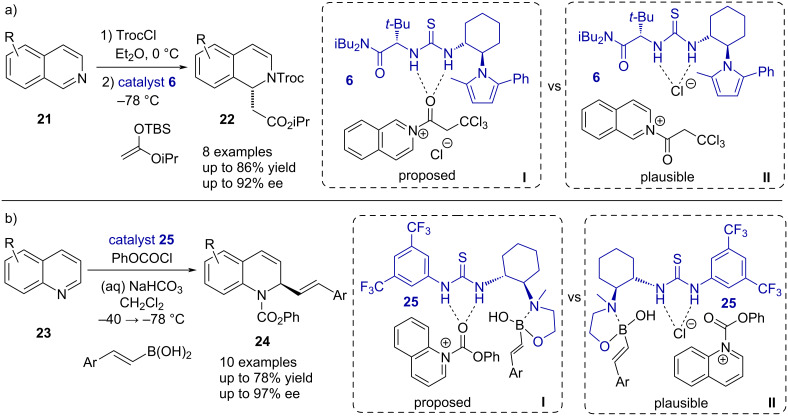
Enantioselective Reissert-type reactions of a) (iso)quinolines with silyl ketene acetals, and b) vinylboronic acids.

During this early period, the group of Jacobsen also reported an asymmetric thiourea-catalyzed Reissert reaction of isoquinolines **21** (Scheme 5a) [36]. The mechanism proceeds by initial activation of the isoquinoline via *N*-acylation and subsequent dearomatization by a nucleophilic attack in the C1 position. Analogously to the Pictet–Spengler cyclization, the group initially speculated that the thiourea catalyst **6** interacts with the carbonyl function of the amide intermediate **I** and, thus, a S_N_2-type mechanism via hydrogen bonding catalysis was proposed. A similar bidentate carbonyl activation proposal was later on reported from the Takemoto group in 2007, where the less reactive quinoline derivatives **23** were employed in a thiourea-catalyzed Reissert reaction (Scheme 5b) [37]. In both cases, however, the binding mode of the catalyst can rather be described by the formation of a close ion pair with the chloride of the *N*-acyl(iso)quinolinium intermediate **II**. Hence, the reaction would follow a S_N_1-type mechanism via anion-binding catalysis. In Jacobsen’s report, the acylating agent 2,2,2-trichloroethyl chloroformate (TrocCl) and nucleophilic silyl ketene acetals were employed to obtain the dihydroisoquinolines **22** in good yields and enantioselectivities up to 92% ee. The Takemoto group with their system also achieved yields up to 78% and enantioselectivities up to 97% ee, using phenyl chloroformate as the acylating reagent and vinylboronic acids as the nucleophiles in the presence of sodium bicarbonate.

The key finding of anion-binding activation opened up a whole new field for asymmetric transformations. Thus, many asymmetric transformations relying on this type of activation mode were subsequently developed [15,23–29]. It is worthy to be mentioned, that Reissert dearomatizations of *N*-heteroarenes, especially of isoquinolines [36], and nucleophilic addition to 1-chloroisochromanes [38] have become benchmark reactions in the context of anion-binding catalysis. Besides reports of thiourea-catalyzed reactions with different nucleophiles [39–40], the focus has also been turned to the development of other catalyst systems that are not based on N–H bonds, such as the chiral silanediol catalysts first reported by Mattson and co-workers in 2013 [19–20]. Furthermore, it is worthy to mention that in parallel to the investigations towards new chiral catalysts and asymmetric methodologies, a few innovative nonchiral alternative H-donor or halide-binding organocatalysts, like, e.g., tridentate phosphoramides [41], onium salts [42] such as Berkessel's pyridinium systems [43], or Huber's bis-iodo imidazolium [44] and neutral bridged 2,6-diiodo‐3,4,5-trifluorophenyl-type catalysts [45]. Additionally, the first asymmetric systems involving purely halogen bond donor catalysis have recently been developed by the groups of Huber [46] and García Mancheño [47]. Moreover, though chloride as halide counter-anion still being particularly prominent, the application of anion-binding catalysis has been successfully demonstrated for other halogens, and different types of substrates such as the benzhydryl cation [48–51].

### Halides as counter-anions vs nucleophiles

**Figure 3:**
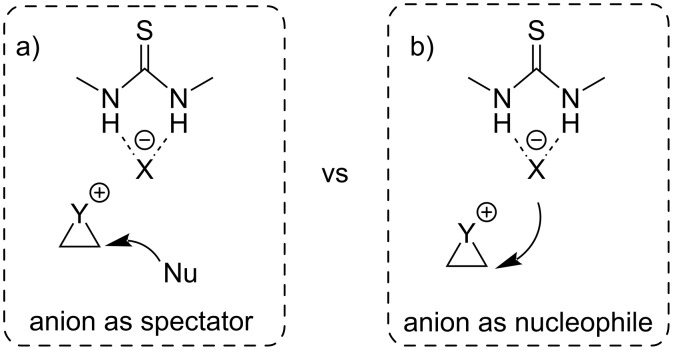
Role of the counter-anion: a) Anion acting as a spectator and b) anion participating directly as the nucleophile.

The latest advances in anion-binding catalysis not only allowed for excellent translation of stereochemical information, but also delivered an insight into the mechanism of the anion-binding process. However, the counter-anion involved, and more precisely the halide anion itself, has remained a mere spectator in the developed catalyses (Figure 3a). Nevertheless, recent reports showed that the bound halide anions can also engage as the nucleophile, which has been exploited in ring opening and related reactions (Figure 3b).

In general, the idea of enantioselective ring opening produces two fixed stereocenters during one synthetic operation, increasing the complexity of the product significantly. This makes asymmetric ring-opening reactions a powerful tool for the synthesis of highly complex target molecules. With this concept in mind, anion-binding catalysis has successfully been employed for asymmetric ring-opening reactions, implying halide anions as both mere counter-anions in the ion-pair complex or active nucleophiles.

**Scheme 6:**
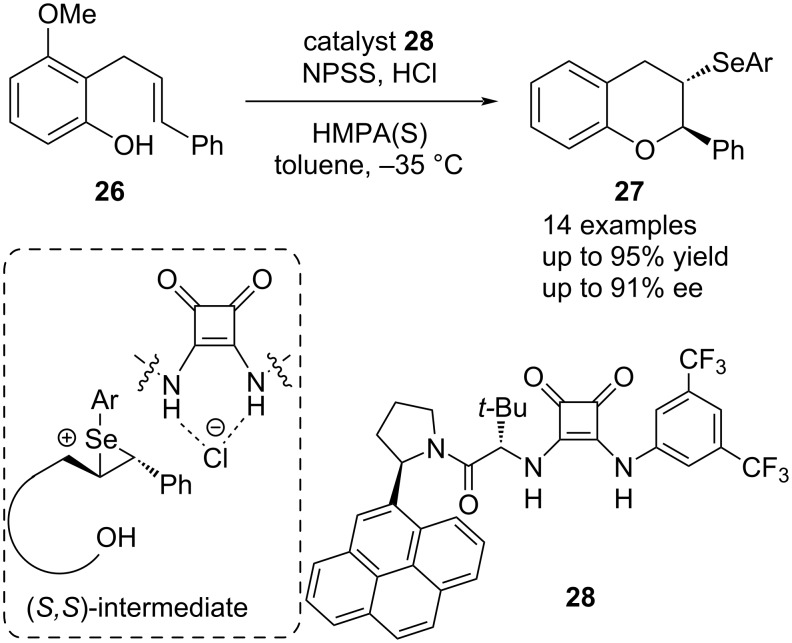
Enantioselective selenocyclization catalyzed by squaramide **28**.

In 2014, Jacobsen et al. developed a highly enantioselective selenocyclization reaction of olefins **26**, using the chiral squaramide **28** as a dual hydrogen bond donor (Scheme 6) [16]. Although early-stage enantio-enrichment during the introduction of selenium is hard to maintain due to the conformational lability of the seleniranium ion [52–54], this initial problem can be exploited through the addition of an anion-binding catalyst. In this way, the configurational scrambling is used for a dynamic kinetic resolution during the intramolecular nucleophilic opening of the seleniranium ring. Through favorable cation–π interactions with the catalyst, the (*S*,*S*)-intermediate reacts faster than its opposing enantiomer, allowing for excellent yields up to 95% and high enantioselectivities up to 91% ee.

**Scheme 7:**
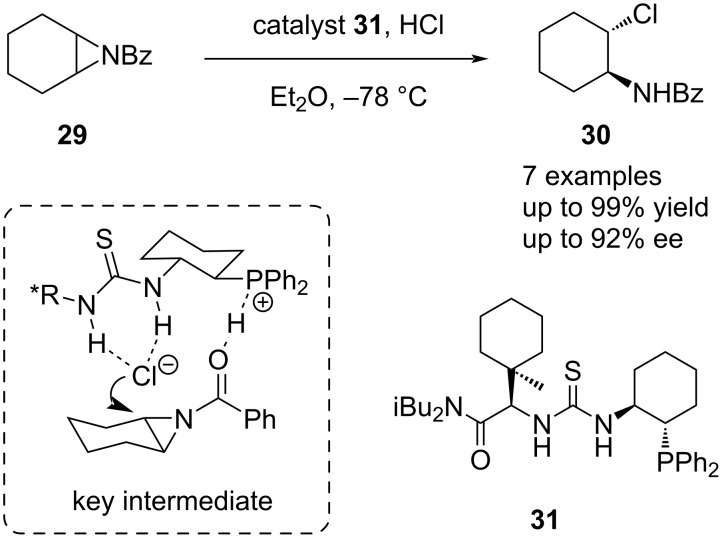
Desymmetrization of *meso*-aziridines catalyzed by bifunctional thiourea catalyst **31**.

In contrast to the previous example, in which the chloride anion was only a spectator linking the substrate and catalyst in the presence of an external nucleophile, halides can also be tuned to participate as the nucleophile in certain reactions. In theory, the close association of the catalyst and the anionic nucleophile might allow for better stereocontrol. An early example utilizing this strategy was provided by Jacobsen and co-workers for the desymmetrization of *meso*-aziridines **29**. In their work, the bifunctional phosphinothiourea catalyst **31** promoted the C–N bond cleavage by hydrochloric acid upon initial protonation (Scheme 7) [55]. Subsequently, the catalyst-bound chloride anion performs a S_N_2-type attack on the coordinated benzoyl-protected aziridine, which leads to a formal addition of HCl.

**Scheme 8:**
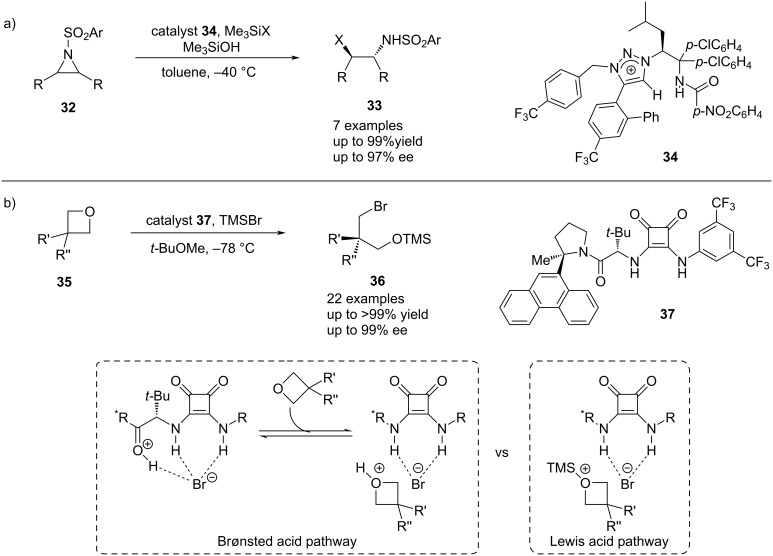
Anion-binding-catalyzed desymmetrization of a) *meso*-aziridines catalyzed by chiral triazolium catalyst **34** by Ooi et al., and b) oxetans catalyzed by chiral squaramide **37** by Jacobsen et al.

This concept was further developed and successfully employed by Ooi in the desymmetrization of *meso*-aziridines **32** with TMSX as chloride and bromide with similar performances as nucleophile precursors using a triazolium-amide chiral catalyst **34** [21] (Scheme 8a), as well as by Jacobsen in the desymmetrization of oxetanes **35** using TMSBr and squaramide **37** as catalyst [56] (Scheme 8b). For the latter, a more detailed mechanistic study was recently provided [57]. The existence of two competing Brønsted acid and Lewis acid mechanistic pathways leading to the same product with high enantioselectivity was then uncovered. Jacobsen et al. reasoned that the key for this highly selective transformation lies in attractive cation–π and cation–dipole secondary interactions between the catalyst and the substrate, which exclusively stabilize the transition state that forms the major enantiomer.

**Scheme 9:**
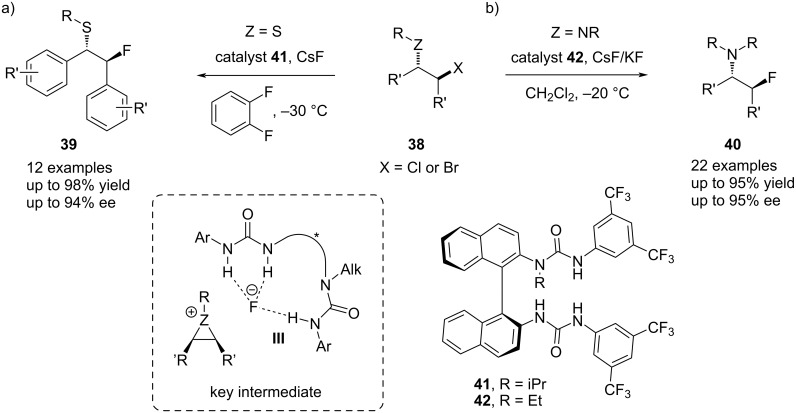
Bis-urea-catalyzed enantioselective fluorination of a) β-bromosulfides and b) β-haloamines by Gouverneur et al.

Furthermore, Gouverneur and co-workers established an enantioselective nucleophilic fluorination protocol using a chiral bis-urea catalyst **41** and CsF as an inorganic fluoride source (Scheme 9a) [18]. By employing in situ-generated *meso*-episulfonium ions, they were able to synthesize β-fluorosulfides **39** in high yields up to 98% and enantioselectivities up to 94% ee. The key step in this transformation is the formation of the noncovalent catalyst–fluoride complex **III** during the phase-transfer step. This provides low amounts of reactive, nucleophilic fluoride in the nonpolar solution, circumventing thereby selectivity and reactivity issues owing to the high basicity of alkali metal fluorides [58–62]. By modifying the reaction conditions, the same group was also able to substitute CsF with KF, making their protocol more cost-effective and widening the scope of the reaction to include β-chloroamines and β-bromoamines as aziridinium precursors **38** (Scheme 9b). In this way, medicinal interesting β-fluoroamines **40** were obtained in good yields and high enantioselectivity up to 95% ee [63].

### Evolution of catalyst designs: from bidentate to supramolecular multidentate anion-binding catalysts

Despite the evident potential that anion-binding catalysis showed in the pioneering publications – especially in regard to exerting high stereocontrol –, the strategy was still faced with typical limiting factors of hydrogen bond donor catalysis, ranging from high catalyst loadings to high dilution, long reaction times and, in some cases, insufficient chirality transfer into the products. As a consequence, many efforts have been spent to overcome those limitations. Some of them rely on the design of more efficient H-donor catalyst structures, offering additional noncovalent interactions in order to provide extra coordination points with the anion, substrate and/or reagent. The most important approaches in this direction used to date are presented in the following.

#### (Thio)urea and squaramide catalysts’ designs

**Scheme 10:**
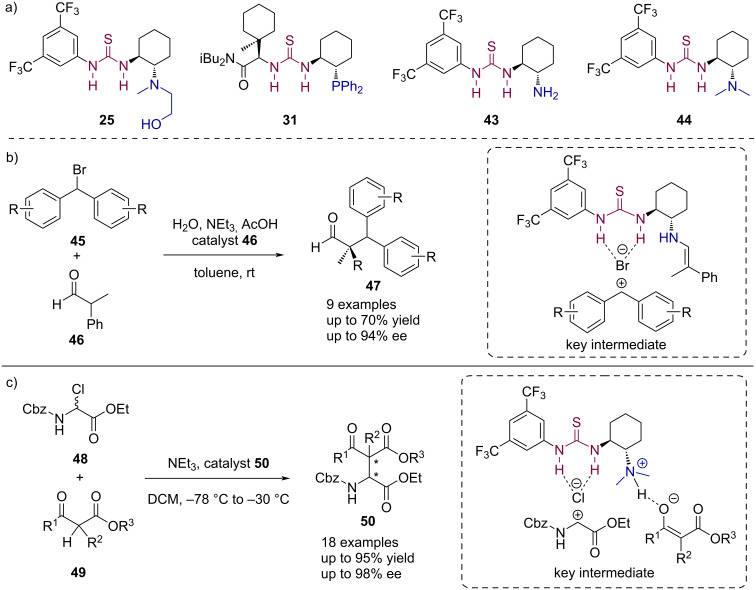
a) Bifunctional thiourea anion-binding – basic/nucleophilic catalysts. Selected applications in b) enantioselective α-alkylation of aldehydes, and c) asymmetric Mannich synthesis of α-amino esters.

**Basic/nucleophilic – H-donor bifunctional catalysts:** Over the past decades, chiral bifunctional catalysts bearing a thiourea as HB-donor and a basic or nucleophilic group such as an amine have emerged as a powerful tool in organocatalysis by assisting to enhance the catalyst performance and fixation of both reaction partners [64–66]. This strategy has also been used in the field of anion-binding catalysis, by designing hydrogen bond donor catalysts with the appropriate additional functionalities in their chiral backbone (Scheme 10a). Some examples have been already presented in the previous sections. For example, catalyst **25** bearing a nucleophilic aminoalcohol functionality interacts with the boronic acid reagent in the Reissert-type reaction with acylated quinolines (Scheme 5b) [36], while the phosphine moiety in the bifunctional phosphinothiourea catalyst **31** allows for heterolytic cleavage of HCl as displayed in Scheme 7 [55].

Moreover, other catalysts with amine functional groups were found more efficient in the enantioselective α-alkylation of aldehydes (Scheme 10b) [48] or in the asymmetric Mannich synthesis of α-amino esters using Takemoto’s bifunctional catalyst **44** [67] (Scheme 10c) described by Jacobsen and co-workers in 2010 and 2014, respectively [50]. In the one hand, while the thiourea unit in catalyst **43** abstracts the bromide in **45** and forms an electrophilic benzhydryl cation, the free amine group activates the aldehyde substrate **46**. The resulting enamine can then serve as the nucleophile as displayed in the key intermediate shown in Scheme 10b. As a result, yields up to 70% and excellent enantioselectivities up to 94% ee could be achieved at room temperature. On the other hand, the secondary amine group in Takemoto’s catalyst **44** acts as a base, abstracting the proton of the enolizable β-ketoester **49** and thus activating the nucleophilic species. This enolate then adds to the cationic substrate from in situ upon halide abstraction of α-chloro amino acid derivatives **48** by the thiourea moiety of the bifunctional catalyst (Scheme 10c, key intermediate), leading to excellent yields and enantioselectivities up to 95% and 98% ee, respectively.

**Cation–π interaction: expanding the functionality of hydrogen bond donor catalysts:** The development of hydrogen bond donor anion-binding catalysts mainly focuses on the interaction and binding properties towards the anionic species. However, the cationic counterpart can have important effects on the kinetics of the systems. This hypothesis has evidently been identified in enzymatic reactions [68]. Mechanistic studies have shown that in such processes, cationic species are stabilized through various attractive interactions with aromatic residues of the enzymes. In fact, these additional stabilizing effects can be exploited in the design of more effective noncovalent catalytic structures for anion-binding catalysis. In this regard, cation–π interactions have been used to develop several types of anion binding-catalyzed transformations such as cyclizations or nucleophilic additions.

**Scheme 11:**
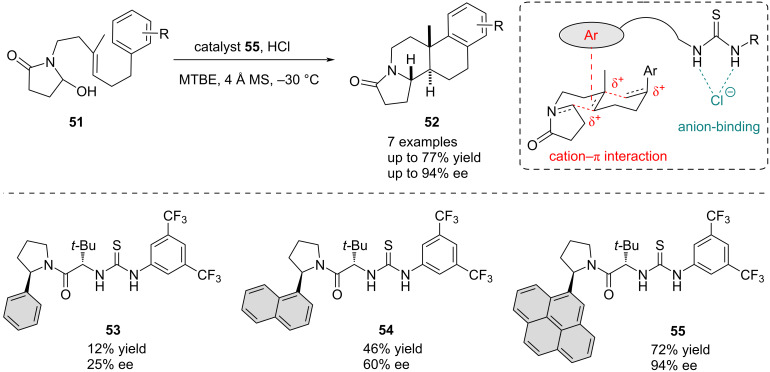
Thiourea-catalyzed enantioselective polycyclization reaction of hydroxylactams **51** through cation–π interaction.

Inspired by cationic terpene-type cyclization cascades, Jacobsen’s group turned their attention to the structure and properties of the chiral part of thiourea catalysts by introducing extended π-groups. A series of thiourea catalysts **53**–**55** with varying aromatic residues were synthesized to elucidate if interactions with the anionic and cationic species could simultaneously be achieved. Hence, in 2010, they successfully showed that such rather small catalysts can mimic nature’s principle of cation–π interactions, allowing for a highly enantioselective polycylization reaction of **51** (Scheme 11) [69]. Modification of the aromatic ring system on the chiral side of the thiourea catalyst proved to be crucial, as both the reactivity and the enantioselectivity were significantly influenced by the stabilization of the cationic substrate and not by interactions with the anion. Specifically, extension of the aromatic system from the simple phenyl (**53**) over the 1-naphthyl (**54**) to the 4-pyrenyl (**55**) substituent led to improved yields from 12% to 72% and enantioselectivities from 25% to 94% ee.

**Scheme 12:**
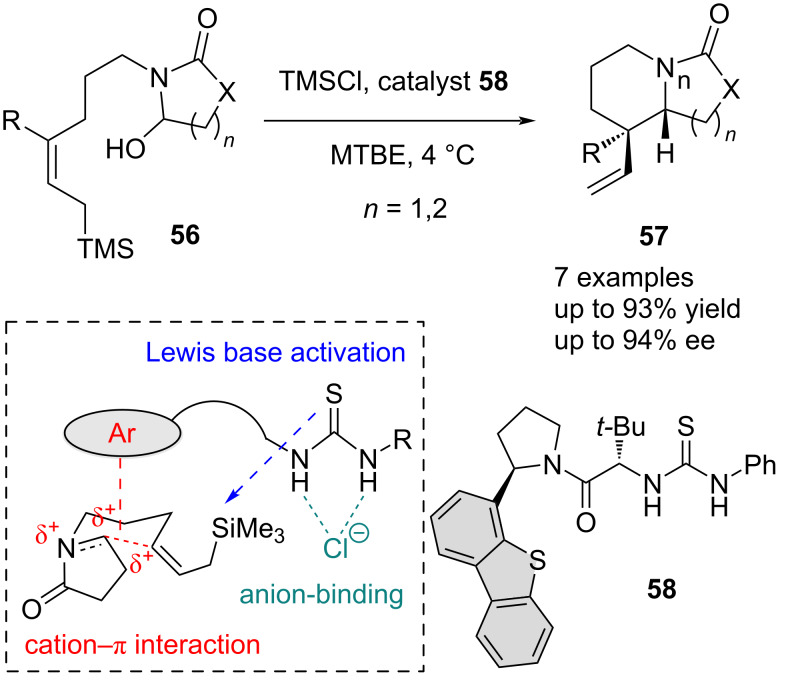
Enantioselective aza-Sakurai cyclization of hydroxylactams **56** implicating additional cation–π and Lewis base activation.

In 2016, this cation–π strategy was further employed for the development of an enantioselective aza-Sakurai cyclization (Scheme 12) [70]. In this transformation, a chiral thiourea catalyst **58** with a dibenzothiophene functionality serves as a dual H-bond donor and Lewis base to facilitate the cyclization of hydroxylactams **56**. Thus, indolizine and quinolidizine frameworks **57** were accessed in excellent yields up to 93% and enantioselectivities up to 94% ee. Increased aromaticity proved again to be essential for achieving high enantioselectivities. Additionally, Lewis base activation of the allylsilane substrates through the thiourea sulfur atom is proposed to be crucial, while the urea analog of the catalysts proved less efficient and led to diminished reactivity and stereoselectivity. Further mechanistic studies corroborated this hypothesis as more electron-rich allylsilane derivatives were consumed slower despite being inherently more nucleophilic.

**Scheme 13:**
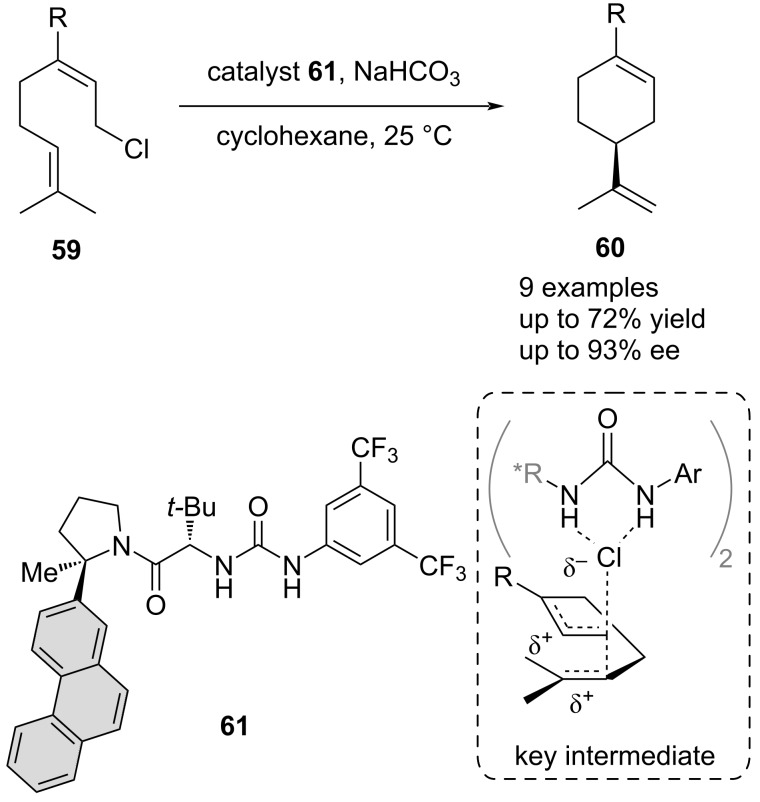
Enantioselective tail-to-head cyclization of neryl chloride derivatives.

Another example highlighting the importance of sidechain catalyst design was given by Jacobsen et al. in the tail-to-head cyclization of neryl chloride and derivatives **59** (Scheme 13) [17]. Mechanistic studies and DFT calculations revealed that an extended π-system in the sidechain of the bidentate urea catalyst **61** was required to form the key aggregate involving two catalyst molecules and the substrate. This complex is the one involved in the rate and enantio-determining ionization step, allowing to furnish the desired products **60** in up to 93% ee.

**Scheme 14:**
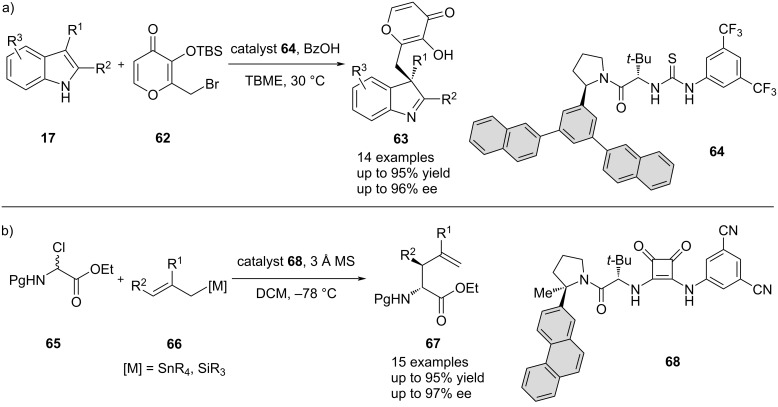
Cation–π interactions in anion binding-catalyzed asymmetric addition reactions: a) addition of indoles to pyrones and b) allylation of α-chloro glycinates.

Finally, similar examples utilizing cation–π interactions have been provided by the group of Jacobsen in the nucleophilic addition of indoles **17** to pyranones **62** (Scheme 14a) [71], as well as in the enantioselective synthesis of α-allyl amino esters **67** by the reaction of α-chloro amino acid derivatives **65** with allyltin and allylsilane **66** nucleophiles [72] (Scheme 14b). In both cases, an extended π-system on the side chain of the chiral thiourea catalysts is able to interact with the reactant and was required to achieve high enantioinductions, providing the corresponding products in excellent yields up to 95% and enantioselectivities up to 96% and 97% ee, respectively.

#### Bis- and macrocyclic thiourea catalysts

**Scheme 15:**
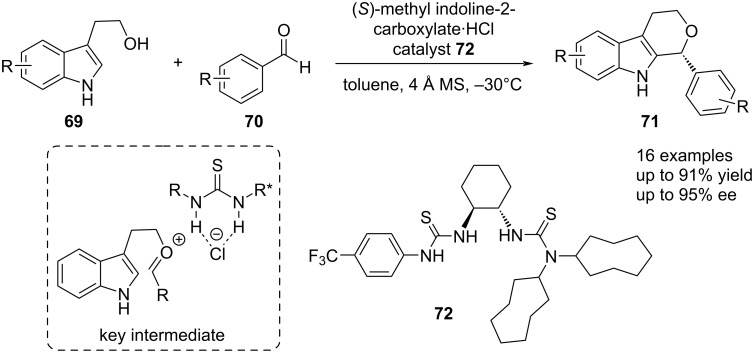
Bisthiourea catalyzed oxa-Pictet–Spengler reaction of indole-based alcohols and aromatic aldehydes under weakly acidic conditions.

Besides the introduction of cation–π interactions in anion-binding catalyst design, bisthiourea catalysts have been applied with the aim of accelerating certain catalytic reactions. In this regard, the group of Seidel reported in 2016 an enantioselective HCl co-catalyzed oxa-Pictet–Spengler reaction employing bisthiourea catalyst **72** bearing two aliphatic groups at one of the nitrogen atoms of one thiourea (Scheme 15) [51]. The key intermediate in this reaction system is the contact ion pair of the thiourea catalyst with the in situ-generated oxycarbenium ion, which enables high enantioselectivities up to 95% ee and yields up to 91%. Furthermore, an investigation of the involved halide counter-anion revealed that chloride was the most potent one in regards of both yield and enantioinduction. Bromine and iodine on the other hand, afforded the final product **71** in lower yields (71% and 90%) and also a detriment in enantioinduction was observed with 76% and 46% ee*,* respectively.

**Scheme 16:**
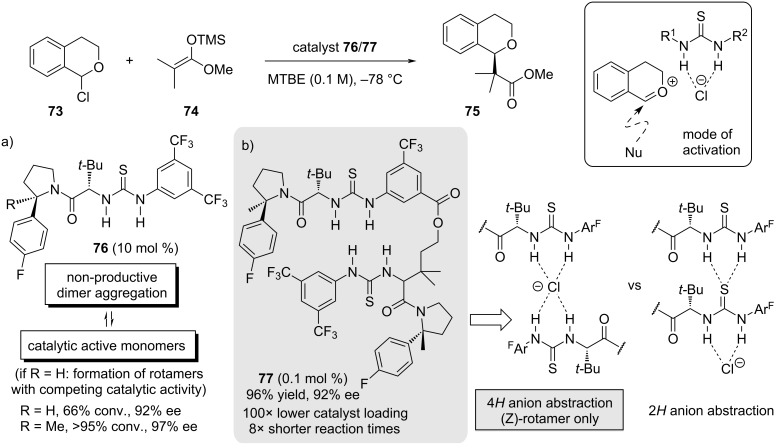
Anion-binding catalyst development in the enantioselective addition of silyl ketene acetals to 1-chloroisochromane (**73**). Limiting factors and influences on catalyst activation and anion abstraction.

Alternatively, Jacobsen´s group carried out a series of studies to elucidate whether the targeted design of a catalyst can increase its efficiency for a given reaction [73–76]. For this purpose, based on their initial findings in 2008 [38], the enantioselective addition of silyl ketene acetals to racemic 1-chloroisochromane (**73**) was more closely examined (Scheme 16) [73–76]. In this type of reaction, thiourea catalyst **76** actively engages in the ionization step by chloride abstraction that leads to the formation of an oxocarbenium intermediate, which then undergoes the stereoselective addition of the nucleophile. Mechanistic insights revealed that two thiourea molecules are, in fact, needed and cooperatively participate in the activation of **73**. However, nonproductive dimeric aggregates form under standard reaction conditions. These dimers exist in different combinations of the thiourea rotamers and lead to competing catalytic pathways (Scheme 16a). Moreover, anion abstraction was calculated to proceed either through a 4*H* abstraction mechanism of two thioureas binding simultaneously to the chloride or through a cooperative 2*H* abstraction mechanism. These findings proved to be decisive in the development of new and more efficient anion-binding catalysts. By introducing a methyl group (R = Me) into the pyrrolidine moiety of the initial catalyst design, the amide is conformationally constricted to the (*Z*)-rotamer [75]. Consequently, improved enantioselectivity and catalytic efficiency could be observed (>95% conv., 97% ee). This design was then further refined by covalently linking two thiourea molecules together to give bis-thiourea catalyst **77** (Scheme 16b) [76]. Due to the linkage of the two molecules, the participating hydrogen bonds are aligned such that a 4*H*-abstraction mode is achieved, which is more likely to ensure higher catalyst activity in the activation step than the competing 2*H*-abstraction pathway. Indeed, with multidentate bis-thiourea catalyst **77**, the catalyst loading could be decreased from 10 to only 0.1 mol % without significant loss of enantioselectivity (96% yield, 92% ee). Ultimately, this work gave a tremendous insight and a myriad of applications of such bis-thiourea catalysts with halogen counter-anions and phosphates [73–78].

**Scheme 17:**
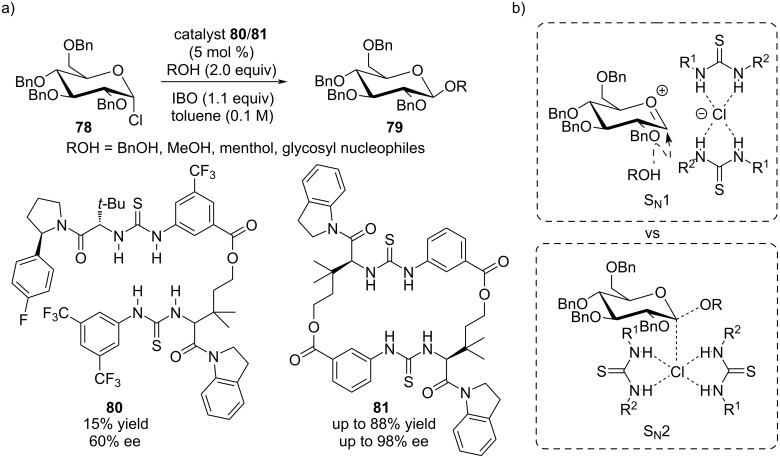
a) Macrocyclic bis-thiourea catalyst in a diastereoselective glycosylation reaction. b) Competing S_N_1 vs S_N_2 reactivity.

Nevertheless, the activation of α-chloro ethers via anion abstraction continued to be a foundation for anion-binding catalyst evolution. In fact, Jacobsen's group further refined the design of their tetradentate N–H-bond donor catalyst **80** by covalently linking it into the more rigid macrocycle **81** (Scheme 17a) [78]. Compared to bis-thiourea **80**, the higher rigidity in the macrocycle **81** not only enforces halide abstraction significantly, but also allowed for a better control of the stereoselectivity in the glycosylation of glycosyl halides **78** with a variety of coupling partners. In this way, the corresponding β-glycosides **79** were almost exclusively obtained (up to 88% yield, up to 98% ee). The reaction was found to proceed stereospecifically with inversion of the anomeric configuration and, therefore, being dependent on the configuration of the electrophilic partner **78**. With this observation, the reaction was concluded to proceed via a S_N_2 mechanism. However, mechanistic investigations revealed the existence of a competing S_N_1 pathway featuring an oxocarbenium cation, which explains the formation of the minor diastereoisomer (Scheme 17b).

#### Non-thiourea-based supramolecular catalysts

**Scheme 18:**
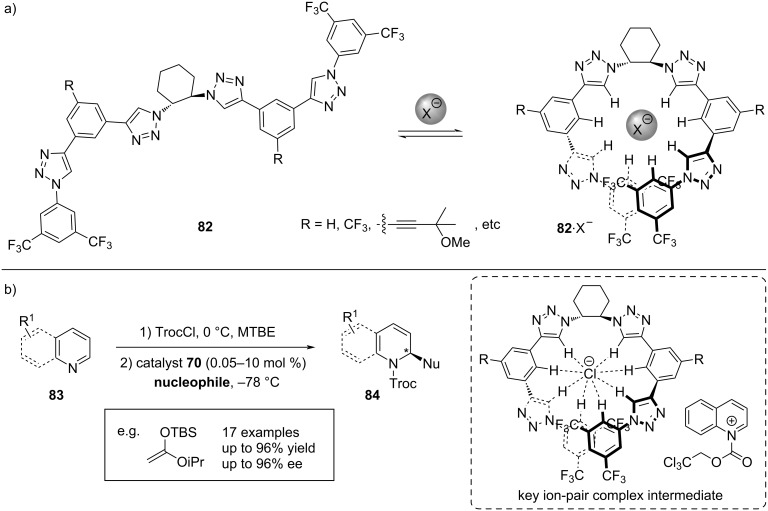
a) Folding mechanism of oligotriazoles upon anion recognition. b) Representative tetratriazole **82** catalyzed enantioselective Reissert-type reaction of quinolines and pyridines with various nucleophiles.

The combination of anion-binding catalysis and supramolecular chemistry is a fairly new arisen field, with a set number of notable examples [79–83]. Next to thioureas, investigations in this area of anion binding were also conducted for other catalytic systems. In 2014, the García group reported a family of chiral helical tetratriazoles **82** as a new class of anion-binding catalysts, which can be considered as supramolecular anion-binding catalysts (Scheme 18) [22]. Not only is the increased H-bonding network in multidentate **82** beneficial for giving a firm control over both regio- and enantioselectivity, but the catalyst itself accommodates the anion by adopting a helical conformation upon complexation (Scheme 18a) [84–86]. Initial studies proved these systems highly effective for the enantioselective Reissert reaction of quinolines with silyl ketene acetals [22], which could be later extended to other *N*- and *O*-heteroarenes and various nucleophiles (Scheme 18b) [87–91].

Computational studies on the helical tetrakistriazole catalyst were additionally carried out, aiming at gaining insight into its interactions with the anion and cationic counterpart of the ionic substrate [86]. Besides the contact to the chloride anion, investigations with tetrabutylammonium chloride (TBACl) and pyridinium chloride salt as model compounds found evidence for productive interactions between the catalyst and the cations. However, these interactions may not solely be attributed to cation–π, but also to cation–H or π–π interactions.

**Scheme 19:**
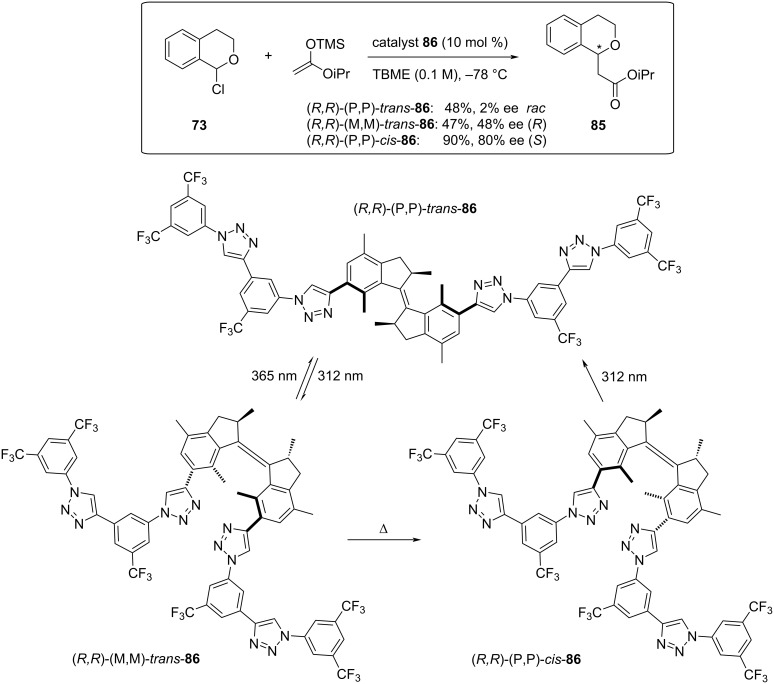
Switchable chiral tetratriazole catalyst **86** in the enantioselective addition of silyl ketene acetals to 1-chloroisochromane.

Some of the advantages of multidentate, supramolecular anion-binding catalysis were recently exploited by the Feringa group, who designed an anion-binding catalyst **86** that fuses the known triazole binding properties with a light-switchable molecular motor. In this way, they were not only able to control the folding of the triazole units through successive irradiation and thermal excitation, but they could also selectively control the stereochemical outcome of the benchmark reaction of 1-chloroisochromane (**73**) with silylketene acetals (Scheme 19) [92].

Such examples, and the advance of anion-binding-catalyzed strategies involving more complex H-bonding networks clearly highlight that it is indeed possible to mimic enzyme-like structures with small-molecule catalysts for asymmetric synthesis.

## Conclusion

In the past two decades, tremendous advances in the field of anion-binding catalysis have been made, evolving as a valuable addition to the synthetic toolbox.

In this review, we have presented the essential role that halide anions, especially chloride, have played in the development of this area of research in the past decades. From the initial endeavors, in which differentiation between classical H-bonding to neutral substrates and the binding to anionic species was delineated, anion-binding interactions became more prominent and started being considered in the design of new syntheses and catalytic approaches. In this context, the emphasis was to display the role of the halide anions and how the predictability of binding properties towards these anions led to the development of a multitude of catalytic concepts and (supramolecular) catalyst systems. Hence, the possibility of employing the catalyst-bound halide anions in the key ion pair complexes as active nucleophiles were also featured. Though less explored so far than their use as simple, inert counter-anions to build the ion pair, this approach provides new possibilities and substantially broadens the synthetic applicability of anion-binding catalysis. Finally, the evolution from simple H-bonding to complex halide anion-binding catalyst designs has been outlined. Recent reports show that synthetic and computational research become more intertwined, and a trend towards multiple noncovalent interactions, as well as supramolecular chemistry, might be in-bound soon.

Based on the tremendous developments in this field thus far, important advances in the understanding of complex anion-binding processes, the design of more potent, efficient catalysts, and the development of innovative activations and reactions can be certainly envisioned to be further evolved in the near future.
